# Phase I study of a biweekly schedule of a fixed dose of cisplatin with increasing doses of paclitaxel in patients with advanced oesophageal cancer

**DOI:** 10.1038/sj.bjc.6690462

**Published:** 1999-06

**Authors:** A van der Gaast, T C Kok, L Kerkhofs, P D Siersema, H W Tilanus, T A W Splinter

**Affiliations:** Medical Oncology; Gastroenterology; Surgery, University Hospital Rotterdam-Dijkzigt, Dr. Molewaterplein 40, 3015 GD Rotterdam, The Netherlands

**Keywords:** oesophageal cancer, cisplatin, paclitaxel, biweekly schedule

## Abstract

We performed this dose-finding study with a fixed dose of cisplatin and increasing doses of paclitaxel given every 2 weeks to determine the maximum tolerable dose of this schedule. Sixty-four patients with advanced oesophageal cancer were treated with a cisplatin dose of 60 mg m^−2^ and increasing doses of paclitaxel from 100 mg m^−2^ up to 200 mg m^−2^ both administered over 3 h for a maximum of six cycles in patients with stable disease or eight cycles in responding patients. Patients were retreated when the granulocytes were > 0.75 × 10^9^ l^−1^ and the platelets > 75 × 10^9^ l^−1^. The dose of paclitaxel could be increased to 200 mg m^−2^ without encountering dose limiting haematological toxicity. At the dose levels 190 mg m^−2^ and 200 mg m^−2^ of paclitaxel cumulative sensory neurotoxicity became the dose-limiting toxicity. The dose intensity of paclitaxel calculated over six cycles rose from 50 mg m^−2^ per week to 85 mg m^−2^ per week. Only three episodes of granulocytopenic fever were encountered out of a total of 362 cycles of treatment. Of the 59 patients evaluable for response, 31 (52%) had a partial or complete response. In a biweekly schedule with a fixed dose of 60 mg m^−2^ cisplatin it is possible to increase the dose of paclitaxel to 180 mg m^−2^. At higher dose levels, neurotoxicity becomes the dose-limiting toxicity. The observed response rate warrants further investigation of this schedule. © 1999 Cancer Research Campaign


*British Journal of Cancer* (1999) **80**(7), 1052–1057
© 1999 Cancer Research Campaign
Article no. bjoc.1998.0462

### Phase I study of a biweekly schedule of a fixed dose of cisplatin with increasing doses of paclitaxel in patients with advanced oesophageal cancer

**A van der Gaast[1], TC Kok[1], L Kerkhofs[1], PD Siersema[2], HW Tilanus[3] and TAW Splinter[1]**

Departments of [1]Medical Oncology, [2]Gastroenterology and [3]Surgery, University Hospital Rotterdam-Dijkzigt, Dr. Molewaterplein 40, 3015 GD Rotterdam, The Netherlands

**Summary** We performed this dose-finding study with a fixed dose of cisplatin and increasing doses of paclitaxel given every 2 weeks to determine the maximum tolerable dose of this schedule. Sixty-four patients with advanced oesophageal cancer were treated with a cisplatin dose of 60 mg m$^{-2}$ and increasing doses of paclitaxel from 100 mg m$^{-2}$ up to 200 mg m$^{-2}$ both administered over 3 h for a maximum of six cycles in patients with stable disease or eight cycles in responding patients. Patients were retreated when the granulocytes were $> 0.75 \times 10^9$ l$^{-1}$ and the platelets $> 75 \times 10^9$ l$^{-1}$. The dose of paclitaxel could be increased to 200 mg m$^{-2}$ without encountering dose limiting haematological toxicity. At the dose levels 190 mg m$^{-2}$ and 200 mg m$^{-2}$ of paclitaxel cumulative sensory neurotoxicity became the dose-limiting toxicity. The dose intensity of paclitaxel calculated over six cycles rose from 50 mg m$^{-2}$ per week to 85 mg m$^{-2}$ per week. Only three episodes of granulocytopenic fever were encountered out of a total of 362 cycles of treatment. Of the 59 patients evaluable for response, 31 (52%) had a partial or complete response. In a biweekly schedule with a fixed dose of 60 mg m$^{-2}$ cisplatin it is possible to increase the dose of paclitaxel to 180 mg m$^{-2}$. At higher dose levels, neurotoxicity becomes the dose-limiting toxicity. The observed response rate warrants further investigation of this schedule.

**Keywords:** oesophageal cancer; cisplatin; paclitaxel; biweekly schedule

The prognosis of patients who present with oesophageal cancer is poor and the majority of patients die within 1 year of diagnosis (Roth et al, 1994; O’Reilly and Forastiere, 1995). Many patients present with locally advanced or metastatic disease, but even those who present with apparently localized disease can often not be cured despite aggressive local treatment. Furthermore, there has been a dramatic increase in the incidence of oesophageal adenocarcinomas (Daly et al, 1996).

Multimodality treatment plays an increasingly important role in patients with oesophageal cancer. Herskovic et al (1992) compared radiation therapy plus chemotherapy with radiation therapy alone. Median survival was prolonged and late relapses were fewer in the patients who received combined therapy. We have conducted a trial comparing surgery plus neoadjuvant chemotherapy with the combination of cisplatin and etoposide followed by surgery in patients with resectable squamous cell carcinoma of the oesophagus, and found that preoperative chemotherapy significantly improved survival (Kok et al, 1997). On the contrary, in the intergroup trial, in which patients with squamous cell carcinomas and adenocarcinomas of the oesophagus were randomized between preoperative chemotherapy with cisplatin and 5-fluorouracil (5-Fu) followed by surgery versus surgery alone, no survival difference was observed between both groups (Kelsen et al, 1997).

*Received 23 June 1998*
*Revised 5 October 1998*
*Accepted 4 December 1998*

*Correspondence to:* A van der Gaast

The treatment for patients with locally advanced, or metastatic, disease is still unsatisfactory. Many phase II trials have been performed in which most chemotherapy regimens consisted of a combination of cisplatin with another agent such as 5-Fu or etoposide (Kok et al, 1996; Kok, 1997). Response rates in the order of 15–40% are usually reported, but the effect on survival remains undetermined.

Paclitaxel has substantial activity in a variety of malignancies, especially ovarian and breast cancer (Holmes et al, 1990; McGuire et al, 1990). At a dose of paclitaxel of 250 mg m$^{-2}$, by 24-h infusion every 3 weeks, a response rate of 32% was reported in 50 evaluable patients with carcinoma of the oesophagus (Ajani et al, 1994). Paclitaxel administered over 3 h in combination with cisplatin and 5-Fu yielded an overall response rate of 45% (Ajani et al, 1996).

Since we are looking for a more active chemotherapy combination than previously used (Kok et al, 1997), which also can be administered as neoadjuvant chemotherapy in the shortest possible period before surgery, we performed a dose-finding study with a fixed dose of cisplatin and escalating doses of paclitaxel given every 2 weeks.

### PATIENTS AND METHODS

This dose-finding study was initiated to determine the toxicities and maximum tolerated dose (MTD) of a combination of paclitaxel and cisplatin given every 2 weeks in patients with metastatic or local-regional unresectable adenocarcinoma, undifferentiated or squamous cell carcinoma of the esophagus or oesophageal–gastric junction area. Eligibility requirements were a life expectancy of 12

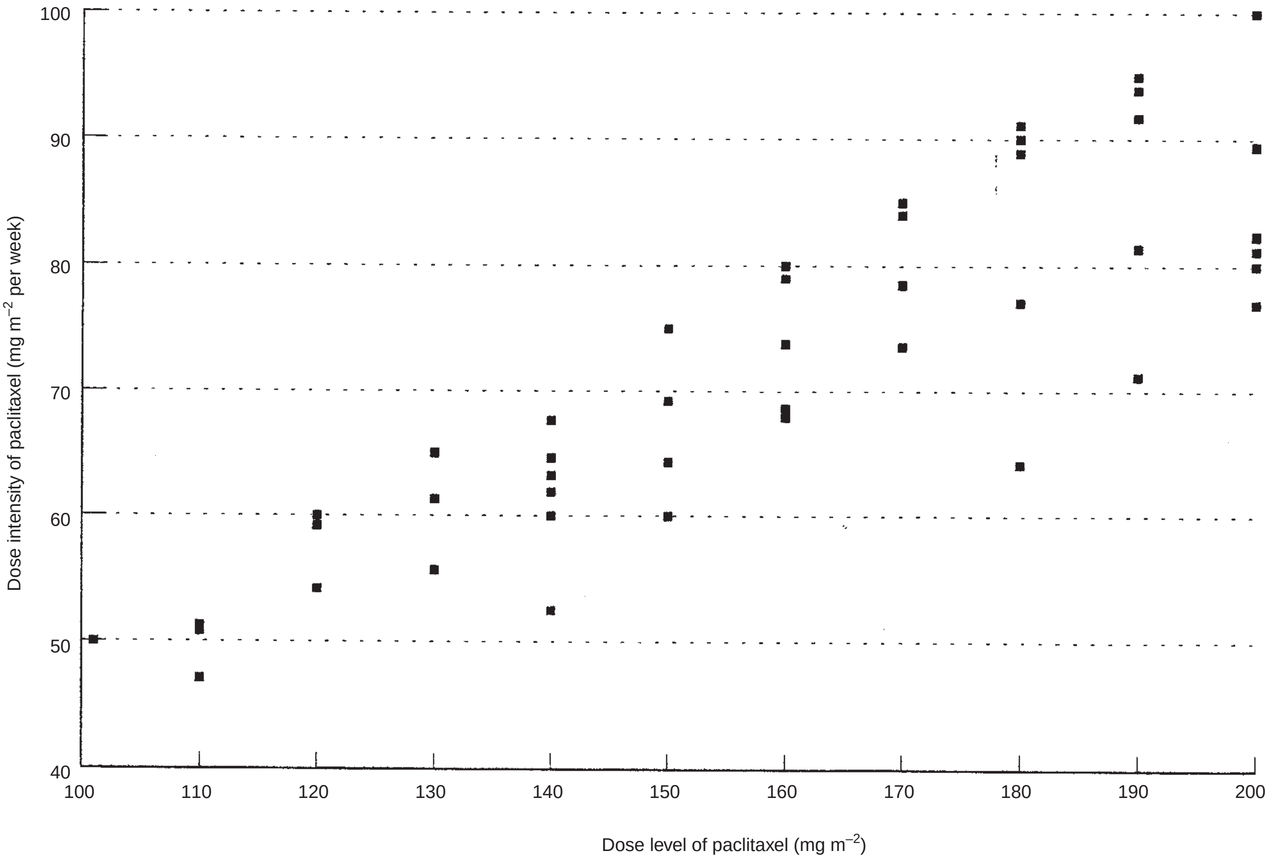

**Figure 1** Dose levels of paclitaxel versus dose intensity weeks or greater; age ≥ 18; ECOG performance status 0, 1, or 2; written and voluntary informed consent; adequate haematological; renal and hepatic functions as defined by: granulocytes $\geq 1.5 \times 10^9$ l$^{-1}$, platelets $\geq 100 \times 10^9$ l$^{-1}$, total bilirubin $\leq 1.5 \times$ upper normal limit and creatinine ≤ 120 μmol l$^{-1}$.

The starting dose of paclitaxel was 100 mg m$^{-2}$ and cisplatin 60 mg m$^{-2}$ given by intravenous (i.v.) infusion every 2 weeks. At subsequent levels, the dose of paclitaxel was increased by 10 mg m$^{-2}$. After prehydration with at least one litre of normal saline, the total calculated dose of paclitaxel, diluted in 500 ml of normal saline, was infused over 3 h. Hereafter the calculated dose of cisplatin was administered over 3 h, followed by post-hydration over 24 h. All patients were premedicated with dexamethasone 20 mg given orally 12 and 6 h prior to the paclitaxel infusion. Thirty minutes before the paclitaxel infusion, the patients received 10 mg dexamethasone, 2 mg clemastine and 50 mg ranitidine, all given i.v. Ondansetron was given as anti-emetic prophylaxis. Patients were retreated when the granulocytes were $> 0.75 \times 10^9$ l$^{-1}$ and the platelets $> 75 \times 10^9$ l$^{-1}$.

Response and toxicity determined the duration of treatment. Patients with stable disease received up to a maximum of six cycles of treatment. In patients achieving a partial or complete response, an additional two cycles were allowed. Treatment was discontinued in patients with disease progression. Toxicity was graded and reported using CTC criteria and response was evaluated using standard WHO criteria. Patients were evaluated for response after the third and sixth course and after discontinuation of therapy. In general, response evaluation was performed by a computerized tomography scan of the chest and upper abdomen, and ultrasonography of the supraclavicular and/or celiac lymph-nodes whenever appropriate. Patients with the primary tumour in situ were also evaluated by endoscopy.

Haematological dose-limiting toxicity was defined as CTC grade III or IV neutropenia with infection or fever requiring parenteral antibiotics, or CTC grade III or IV thromocytopenia requiring two or more platelet transfusions within one cycle, or resulting in ≥ CTC grade 2 haemorrhage. Non-haematological dose-limiting toxicity was defined as CTC grade III or IV non-haematological toxic effects, with the exception of nausea and emesis. Dose reduction/treatment delay dose-limiting toxicity was defined as dose reductions and/or treatment delay for ≥ 1 week for reasons of toxicity. The MTD was reached if ≥ two of three patients had treatment delay dose-limiting toxicity during the first two cycles at a given dose level. If dose-limiting toxicity of any type was seen in one of three patients within the first two cycles at a given level, three more patients were enrolled at this dose level. If ≥ two of six patients experienced dose-limiting toxicity, that dose level was considered the maximum tolerated dose. Patient characteristics are listed in Table 1.

### RESULTS

Fifty-eight of the 64 patients entered into this study received at least three cycles of treatment. The reasons for fewer treatment

*© 1999 Cancer Research Campaign*

**Table 1** Patients' characteristics

| Characteristic | No. of patients | (%) |
|---|---|---|
| Total patients | 64 | |
| Sex | | |
| Female | 17 | (27) |
| Male | 47 | (73) |
| Age, years | | |
| Median | 56 | |
| Range | 37–74 | |
| Performance status (Karnofsky) | | |
| 60% | 2 | (3) |
| 70% | 8 | (12) |
| 80% | 12 | (19) |
| 90% | 32 | (50) |
| 100% | 9 | (14) |
| Unknown | 1 | (2) |
| Histology | | |
| Adenocarcinoma | 33 | (52) |
| Squamous cell carcinoma | 30 | (47) |
| Undifferentiated carcinoma | 1 | (1) |
| Extent of disease | | |
| Locally advanced/unresectable | 13 | (20) |
| Primary with distant metastases | 41 | (64) |
| Metastases after prior resection | 10 | (16) |
| Metastatic sites | | |
| Supraclavicular lymph nodes | 16 | (25) |
| Celiac lymph nodes | 26 | (41) |
| Liver | 7 | (11) |
| Bone | 2 | (3) |
| Mediastinal recurrence | 4 | (6) |
| Other | 15 | (23) |

cycles were the following: one patient died of upper gastro-intestinal bleeding without thrombocytopenia 8 days after the start of chemotherapy; one patient's condition deteriorated after the first course of chemotherapy because of an aspiration pneumonia, and further treatment was withheld; one patient developed signs of spinal cord compression due to bone metastases a few days after start of chemotherapy, was treated with radiotherapy and went off study; one patient died of ruptured aortic aneurysm 21 days after start of chemotherapy; one patient had disease progression after one cycle; and one patient developed a cholestatic hepatitis after two cycles, most probably due to a combination of amoxicillin and clavulanic acid. This treatment had been prescribed because of respiratory infection before start of chemotherapy.

The paclitaxel dose was escalated from 100 mg m$^{-2}$ to 200 mg m$^{-2}$ and a total of 362 cycles of treatment were administered with a median number of six cycles (range 1–8). Haematological dose-limiting toxicity was not reached. Sixty-nine per cent of the patients received at least six cycles of treatment.

Sixty-one chemotherapy cycles were delayed in 33 (57%) of the 58 patients who received at least three cycles of chemotherapy. Forty-five cycles in 24 patients were delayed for a maximum of 1 week because of a granulocyte count $< 0.75 \times 10^9$ l$^{-1}$. The details of the treatment delays are shown in Table 2. A dose reduction was applied in one patient at the dose level 200 mg m$^{-2}$ of paclitaxel because of granulocytopenic fever in the preceding course. The achieved dose intensity of paclitaxel in milligrams per square metre per week (mg m$^{-2}$ per week), calculated over six cycles per dose level, is shown in Figure 1.

The frequency of grade 3 and 4 leucocytopenia and granulocytopenia only slightly increased at the higher dose levels. Three patients experienced granulocytopenic fever. The first patient mentioned earlier, who was treaded with radiotherapy after the start of chemotherapy, was admitted with granulocytopenic fever 14 days after start of chemotherapy. The other two patients were treated at dose level of 200 mg m$^{-2}$ paclitaxel and had granulocytopenic fever after the first and third cycles respectively. All three patients recovered. Grade 1 thrombocytopenia was observed in five patients at paclitaxel dose levels of 140 mg m$^{-2}$ (one patient), 150 mg m$^{-2}$ (one patient), 180 mg m$^{-2}$ (one patient) and 200 mg m$^{-2}$ (two patients). Two patients had grade 2 thrombocytopenia at the dose levels of 130 and 140 mg m$^{-2}$ of paclitaxel. No grade 3 or 4 thrombocytopenia was observed at any dose level. The worst haematological toxicity per dose levels for all patients who received at least three cycles of treatment is listed in Table 3.

The neurotoxicity per dose level is presented in Table 4. A clear increase in the neurotoxicity can be observed at the higher dose levels. Almost all these patients had a neuro-sensory toxicity characterized by paraesthesiae and sensory loss sometimes interfering with functioning. Frequently, the onset or worsening of the neurotoxicity occurred after treatment had been stopped and was only partially reversible. At the dose level of 200 mg m$^{-2}$ of paclitaxel in five patients' treatment was discontinued after the fourth or fifth cycle because of grade 2 or 3 neuroxtoxicity.

**Table 2** Treatment delays due to granulocytopenia during the first six cycles in patients who received at least three cycles of chemotherapy

| Paclitaxel dose (mg m$^{-2}$) | No. of patients | No. of patients with a delay | No. of courses with a delay | Total no. of courses (% delayed) |
|---|---|---|---|---|
| 100 | 3 | 0 | 0 | 18 (0%) |
| 110 | 3 | 1 | 2 | 18 (11%) |
| 120 | 3 | 0 | 0 | 17 (0%) |
| 130 | 3 | 2 | 3 | 18 (17%) |
| 140 | 6 | 4 | 8 | 36 (22%) |
| 150 | 7 | 3 | 6 | 35 (17%) |
| 160 | 6 | 3 | 5 | 36 (14%) |
| 170 | 6 | 2 | 3 | 33 (9%) |
| 180 | 6 | 2 | 5 | 35 (14%) |
| 190 | 5 | 2 | 5 | 30 (17%) |
| 200 | 10 | 5 | 8 | 45 (18%) |

 *© 1999 Cancer Research Campaign*

**Table 3** Worst grade of leucocytopenia per dose level

| Paclitaxel dose ($mg\ m^{-2}$) | No. of patients | Grade | | | | |
|---|---|---|---|---|---|---|
| | | 0 | 1 | 2 | 3 | 4 |
| | | Leucocytes (granulocytes) | | | | |
| 100 | 3 | 1 (0) | 1 (1) | 1 (0) | 0 (1) | 0 (1) |
| 110 | 3 | 0 (0) | 1 (1) | 2 (0) | 0 (1) | 0 (1) |
| 120 | 3 | 1 (0) | 0 (1) | 2 (0) | 0 (2) | 0 (0) |
| 130 | 3 | 0 (0) | 1 (0) | 2 (0) | 0 (1) | 0 (2) |
| 140 | 6 | 0 (0) | 1 (0) | 3 (0) | 2 (3) | 0 (3) |
| 150 | 7 | 1 (0) | 0 (1) | 4 (1) | 2 (1) | 0 (4) |
| 160 | 6 | 1 (0) | 1 (1) | 3 (3) | 2 (1) | 0 (2) |
| 170 | 6 | 1 (0) | 1 (2) | 3 (1) | 1 (2) | 0 (1) |
| 180 | 6 | 0 (0) | 2 (1) | 2 (1) | 2 (3) | 0 (1) |
| 190 | 5 | 1 (1) | 2 (1) | 2 (0) | 0 (2) | 0 (1) |
| 200 | 10 | 2 (1) | 1 (0) | 4 (2) | 2 (1) | 1 (6) |

**Table 4** Worst grade neurotoxicity per dose level

| Paclitaxel dose ($mg\ m^{-2}$) | No. of patients | Grade | | | | |
|---|---|---|---|---|---|---|
| | | 0 | 1 | 2 | 3 | 4 |
| 100 | 3 | 2 | 0 | 1 | 0 | 0 |
| 110 | 3 | 3 | 0 | 0 | 0 | 0 |
| 120 | 3 | 2 | 1 | 0 | 0 | 0 |
| 130 | 3 | 2 | 1 | 0 | 0 | 0 |
| 140 | 6 | 4 | 2 | 0 | 0 | 0 |
| 150 | 7 | 3 | 2 | 1 | 1 | 0 |
| 160 | 6 | 1 | 4 | 1 | 0 | 0 |
| 170 | 6 | 4 | 1 | 1 | 0 | 0 |
| 180 | 6 | 2 | 4 | 0 | 0 | 0 |
| 190 | 5 | 1 | 1 | 1 | 2 | 0 |
| 200 | 10 | 0 | 1 | 4 | 5 | 0 |

**Table 5** Worst grade other toxicities (CTC) at all dose levels (minimum number of courses = 3)

| | No. of patients | Grade | | | | |
|---|---|---|---|---|---|---|
| | | 0 | 1 | 2 | 3 | 4 |
| Haemoglobin | 58 | 9 | 25 | 22 | 2 | |
| Alopecia | 58 | 2 | 1 | 55 | | |
| Nausea | 58 | 11 | 23 | 21 | 3 | |
| Vomiting | 58 | 26 | 14 | 10 | 8 | |
| Mucositis | 58 | 51 | 7 | | | |
| Nephrotoxicity | 58 | 55 | | 1 | 2 | |
| Myalgia | 58 | 20 | 25 | 13 | | |
| Fatigue | 58 | 21 | 22 | 14 | 1 | |

The other toxicities were usually mild and easily manageable, and not substantially worse at the higher dose levels. A summary of these toxicities is listed in Table 5. Two patients had grade 3 nephrotoxicity after the sixth and eighth cycle. The first patient was admitted 2 weeks after the sixth cycle because of an obstructive uropathy complicated by a urosepsis without leucocytopenia or granulocytopenia. Despite intensive treatment, the patient died. The second patient had a reversible impairment of the renal function after the eighth cycle, which was most probably due to the use of a non-steroidal anti-inflammatory agent.

Fifty-nine patients were evaluable for response. Two patients (3%) had a complete response with a duration of 5 and 7 months. Twenty-nine patients (49%) achieved a partial response with a median duration of 7 months (range 3–16+ months). Eighteen patients (31%) had stable disease with a median duration of 5 months (range 3–11+ months) and ten patients (17%) progressed. Seventeen of the 29 patients (59%) with an adenocarcinoma had a partial response, and 14 of the 29 patients (48%) with a squamous cell carcinoma, had a partial or complete response. Responses were observed at all dose levels and there was no indication for a dose–response relationship.

*© 1999 Cancer Research Campaign*

### DISCUSSION

Paclitaxel either given as a single agent or in combination with cisplatin is usually administered once every 3 weeks. However, since the period of neutropenia is usually brief, shorter inter-treatment intervals may be possible to increase dose density. Studies with weekly or biweekly paclitaxel have been reported (Parimoo et al, 1996; Fennelly et al, 1997).

In two phase I studies, a fixed dose of cisplatin of 60 mg $m^{-2}$ and an escalating dose of paclitaxel by 3-h infusion in a biweekly schedule was tested (Gelmon et al, 1996*a*; Swenerton et al, 1996). In the study of Swenerton et al (1996) granulocytopenia, which prevented retreatment at the scheduled time, was the dose-limiting toxicity at a paclitaxel dose level of 120 mg $m^{-2}$. Two of the six patients had granulocytopenia ($< 0.75 \times 10^9$ $l^{-1}$) on day 14 of the first cycle at this dose level. In the study of Gelmon et al (1996*a*) dose-limiting neutropenia was seen at a paclitaxel dose of 100 mg $m^{-2}$. The latter study included 27 patients with metastatic breast cancer, most of whom had received prior adjuvant chemotherapy. In the subsequent phase II study with paclitaxel 90 mg $m^{-2}$ and cisplatin 60 mg $m^{-2}$, responses were observed in 85% of the assessable patients (Gelmon et al, 1996*a*). In two other phase II trials of biweekly paclitaxel by 3-hour infusion and cisplatin in advanced breast cancer using the same dose–response, rates of 23% and 60% were observed in 13 and 25 patients respectively (McCaskill-Stevens et al, 1996; Sparano et al, 1997).

Sørensen et al (1997) reported a response rate of 43% in 40 evaluable patients with non-small-cell lung cancer using a biweekly schedule of cisplatin 60 mg $m^{-2}$ and a 3-h infusion of paclitaxel 110 mg $m^{-2}$. In a phase I/II study in patients with non-small-cell lung cancer of biweekly paclitaxel and a fixed dose of 60 mg $m^{-2}$ cisplatin, the dose of paclitaxel could be escalated to 140 mg $m^{-2}$ without dose-limiting toxicity (Gelmon et al, 1996*b*). Such data suggest that the dose-limiting toxicity may be dependent on patient selection and/or tumour type, or other presently unknown factors.

In our study, it was possible to increase the dose of paclitaxel to 200 mg $m^{-2}$ in combination with 60 mg $m^{-2}$ cisplatin. Haematological dose-limiting toxicity was not observed. Surprisingly the percentage of treatment delays due to granulocytopenia did not substantially increase with the higher dosages of paclitaxel. The dose intensity of paclitaxel calculated over 6 cycles rose from 50 mg $m^{-2}$ per week to approximately 85 mg $m^{-2}$ per week. Only three episodes of granulocytopenic fever were observed in three patients. This suggests that retreatment is safe when the granulocyte count is $> 0.75 \times 10^9$ $l^{-1}$.

Cumulative sensory neuropathy became the dose-limiting toxicity and was the reason that, at the dose level of 200 mg $m^{-2}$, 50% of the patients did not complete the planned six cycles of treatment. Also, at the dose level of 190 mg $m^{-2}$ two patients eventually developed a grade 3 neuropathy. Neuropathy caused by paclitaxel is related to the absolute dose of paclitaxel administered during each course, and the cumulative dose (Rowinsky et al, 1993). Although the severity of the peripheral neuropathy is correlated with paclitaxel $C_{ss}$, the absolute dose administered during each course seems to be equally predictive for the development of neuropathy. Connelly et al (1996) suggested potentiation of paclitaxel's neuropathy by cisplatin when paclitaxel was given over 3 h rather than over 24 h. However, a disadvantage of a 24-h infusion duration of paclitaxel instead of a 3-h infusion duration is the occurrence of more profound myelosuppression (Eisenhauer et al, 1994). It is unclear whether a treatment interval of 2 weeks instead of 3 weeks between courses also influences the severity of neuropathy caused by the combination of cisplatin and paclitaxel. Hilkens et al (1995) found no increase in neurotoxicity with more intensive dosing schedules of cisplatin within a cumulative dose range of 280–675 mg $m^{-2}$. At the higher dose levels, we found that the neuropathy often had a rapid onset, as has also been recently reported in a phase I dose-escalating study with paclitaxel administered over 3 h and cisplatin administered over 4 h in a 3-weekly schedule (Gordon et al, 1997).

Other toxicities were usually mild and the observed response rate of 52% in 59 evaluable patients is a very promising result in this patient group.

In conclusion, in a biweekly schedule with a fixed dose of 60 mg $m^{-2}$ cisplatin, it is possible to increase the dose of paclitaxel to 200 mg $m^{-2}$ without encountering haematological dose-limiting toxicity. At the dose levels 190 mg $m^{-2}$ and 200 mg $m^{-2}$ of paclitaxel, grade 3 neurotoxicity was frequently seen and was the reason that, at the last dose level, 50% of the patients had to stop treatment prematurely. Consequently, 180 mg $m^{-2}$ paclitaxel in combination with 60 mg $m^{-2}$ cisplatin seems to be the maximum tolerable dose. The observed response rate in this study certainly warrants further investigation of this biweekly schedule in patients with oesophageal cancer.

### ACKNOWLEDGEMENT

This study was supported by a grant from Bristol-Meyers-Squibb.

### REFERENCES

Ajani JA, Ilson DH, Daugherty K, Pazdur R, Lynch PM and Kelsen DP (1994) Activity of taxol in patients with squamous cell carcinoma and adenocarcinoma of the esophagus. *J Natl Cancer Inst* **86**: 1086–1091

Ajani JA, Ilson DH and Kelsen DP (1996) Paclitaxel in the treatment of patients with upper gastrointestinal carcinomas. *Semin Oncol* **23**: 55–58

Connelly E, Markman M, Kennedy A, Webster K, Kulp B, Peterson G and Belinson J (1996) Paclitaxel delivered as 3-hr infusion with cisplatin in patients with gynecologic cancers: unexpected incidence of neurotoxicity. *Gynecol Oncol* **62**: 166–168

Daly JM, Karnell L and Menck HR (1996) National Cancer Data Base report on esophageal carcinoma. *Cancer* **78**: 1820–1828

Eisenhauer EA, ten Bokkel Huinink WW, Swenerton KD, Gianni L, Myles J, van der Burg MEL, Kerr I, Vermorken JB, Buser K, Colombo N, Bacon M, Santabarbara P, Onetto N, Winograd W and Canetta R (1994) European-Canadian randomized trial of paclitaxel in relapsed ovarian cancer: high-dose versus low-dose and long versus short infusion. *J Clin Oncol* **12**: 2654–2666

Fennelly D, Aghajanian C, Shapiro F, O'Flaherty C, McKenzie M, O'Connor C, Tong W, Norton L and Spriggs D (1997) Phase I and pharmacologic study of paclitaxel administered weekly in patients with relapsed ovarian cancer. *J Clin Oncol* **15**: 187–192

Gelmon KA, O'Reilly SE, Tolcher AW, Campbell C, Bryce C, Ragaz J, Coppin C, Plenderleith IH, Ayers D, McDermott B, Nakashima L, Healy D and Onetto N (1996*a*) Phase I/II trial of bi-weekly paclitaxel and cisplatin in the treatment of metastatic breast cancer. *J Clin Oncol* **14**: 1185–1191

Gelmon KA, Murray N, Melosky B, Shah A, Page R, Dulude H and Rielly S. (1996*b*) Phase I/II study of biweekly paclitaxel and cisplatin in non-small-cell lung cancer. *Proc Am Soc Clin Oncol* **15**: 405 (Abstract)

Gordon AN, Stringer CA, Matthews CM, Willis DL and Nemunaitis J (1997) Phase I dose escalation of paclitaxel in patients with advanced ovarian cancer receiving cisplatin: rapid development of neurotoxicity is dose-limiting. *J Clin Oncol* **15**: 1965–1973

Herskovic A, Martz K, al-Sarraf M, Leichman L, Brindle J, Vaitkevicius V, Cooper J, Byhardt R, Davis L and Emami B (1992) Combined chemotherapy and radiotherapy compared with radiotherapy alone in patients with cancer of the esophagus. *N Engl J Med* **326**: 1593–1598

*© 1999 Cancer Research Campaign*

Hilkens PH, van der Burg ME, Moll JW, Planting AS, van Putten WL, Vecht CJ and van den Bent MJ (1995) Neurotoxicity is not enhanced by increased dose intensities of cisplatin administration. *Eur J Cancer* **31**: 678–681

Holmes FA, Walters RS, Theriault RL, Forman AD, Newton LK, Raber MN, Buzdar AU, Frye DK and Hortobagyi GN (1990) Phase II trial of Taxol, an active drug in the treatment of metastatic breast cancer. *J Natl Cancer Inst* **82**: 1247–1249

Kelsen D, Ginsberg R, Qian C, Gunderson L, Mortimer J, Estes N, Hailer D, Ajani J, Kocha W, Roth J and Minsky B (1997) Chemotherapy followed by operation versus operation alone in the treatment of patients with localized esophageal cancer: a preliminary report of intergroup study 113 (RTOG 89-11). *Proc Am Soc Clin Oncol* **16**: 276 (Abstract)

Kok TC, van der Gaast A, Stoter G, Tilanus H, Eykenboom W, Dees J, van Overhagen H and Splinter T (1996) Cisplatin and etoposide in esophageal cancer. A phase II study. *Br J Cancer* **74**: 980–984

Kok TC (1997) Chemotherapy in esophageal cancer: a review. *Cancer Treat Rev* **23**: 65–85

Kok TC, van Lanschot J, Siersema PD, van Overhagen H and Tilanus HW (1997) Neoadjuvant chemotherapy in operable esophageal squamous cell cancer: final report of a phase III multicenter randomized controlled trial. *Proc Am Soc Clin Oncol* **16**: 277 (Abstract)

McCaskill-Stevens W, Ansari R, Fisher W, Pennington K, Dobbs C, Gonin R, Schaefer S, Loesch D and Sledge G (1996) Phase II study of biweekly cisplatin and paclitaxel in the treatment of metastatic breast cancer. *Proc Am Soc Clin Oncol* **15**: 120 (Abstract)

McGuire WP, Hoskins WJ, Brady MF, Kucera PR, Partridge EE, Look KY, Clarke-Pearson DL and Davidson M (1996) Cyclophosphamide and cisplatin compared with paclitaxel and cisplatin in patients with stage III and IV ovarian cancer. *N Engl J Med* **334**: 1–6

O'Reilly S and Forastiere A (1995) Is surgery necessary with multimodality treatment of esophageal cancer? *Ann Oncol* **6**: 519–521

Parimoo D, Garcia A, Muggia F, Dimery I, Rogers M and Jeffers S (1996) Tolerance of paclitaxel (Taxol) 3-hr infusion with G-CSF on a biweekly schedule. *Proc Am Soc Clin Oncol* **15**: 181 (Abstract)

Roth JA and Putnam JB Jr (1994) Surgery for cancer of the esophagus. *Sem Oncol* **21**: 453–461

Rowinsky EK, Chaudhry V, Forastiere A, Sartorius SE, Ettinger DS, Grochow LB, Lubejko BG, Cornblath DR and Donehower RC (1993) Phase I and pharmacologic study of paclitaxel and cisplatin with granulocyte colony-stimulating factor: neuromuscular toxicity is dose-limiting. *J Clin Oncol* **11**: 2010–2020

Sørenson JB, Wedervang K and Dombernowsky P (1997) Preliminary results of a phase II study of paclitaxel and cisplatin in patients with non-small cell lung cancer. *Semin Oncol* **24**: S12.18–S12.20

Sparano JA, Neuberg D, Glick JH, Robert NJ, Goldstein LJ, Sledge GW and Wood W (1997) Phase II trial of bi-weekly paclitaxel and cisplatin in advanced breast carcinoma: an Eastern Cooperative Oncology Group study. *J Clin Oncol* **15**: 1880–1004

Swenerton K, Hoskins P, Stuart G, Batist G, Pike J, Onetto N, Fisher B and Eisenhauer E (1996) A phase I study of biweekly paclitaxel/cisplatin as initial therapy for advanced ovarian cancer. A study of the National Cancer Institute of Canada Clinical Trials Group. *Ann Oncol* **7**: 1077–1079

*© 1999 Cancer Research Campaign*
